# Investigating the zoonotic origins of ESBL-producing *E. coli* in community-acquired urinary tract infections in Ecuador

**DOI:** 10.1128/spectrum.03325-25

**Published:** 2026-04-08

**Authors:** Yashan Wang, Daniel E. Park, Maliha Aziz, Liseth Salinas, Edward Sung, Søren Hallstrøm, Cindy M. Liu, Marc Stegger, Zhenke Wu, Gabriel Trueba, Joseph N. S. Eisenberg, Jay P. Graham, Lance B. Price

**Affiliations:** 1 Department of Environmental and Occupational Health, Antibiotic Resistance Action Center, The George Washington University 8367, Washington, DC, USA; 2 Colegio de Ciencias Biológicas y Ambientales, Instituto de Microbiología, Universidad San Francisco de Quito 27902, Quito, Ecuador; 3 Department for Sequencing and Bioinformatics, Statens Serum Institut 4326, Copenhagen, Denmark; 4 Antimicrobial Resistance and Infectious Diseases Laboratory, Harry Butler Institute, Murdoch University 5673, Murdoch, Western Australia, Australia; 5 Department of Biostatistics, School of Public Health, University of Michigan 1259, Ann Arbor, Michigan, USA; 6 Department of Epidemiology, School of Public Health, University of Michigan 1259, Ann Arbor, Michigan, USA; 7 Division of Environmental Health Sciences, School of Public Health, University of California 1438, Berkeley, California, USA; Universidade de Sao Paulo, São Paulo, Brazil

**Keywords:** ESBL-producing *E. coli*, poultry, urinary tract infection, foodborne, *Escherichia coli*, Ecuador, domestic animals, backyard animals

## Abstract

Extended-spectrum β-lactamase-producing *Escherichia coli* (ESBL-producing *E. coli*) pose a growing global health threat. Although Latin America has been identified as a global hotspot of antimicrobial resistance, the zoonotic contribution to drug-resistant infections in the region remains poorly defined. We analyzed 137 clinical ESBL-producing *E. coli* isolates from urinary tract infections (UTIs) in Quito, Ecuador, applying a Bayesian latent class model informed by host-associated mobile genetic elements to estimate the fraction of infections attributable to food-animal sources. We estimated that 25.5% (35/137) of UTI isolates were putative zoonotic cases. This proportion rose to 42.5% after excluding ST131-*H*30, a human-associated pandemic lineage. Putative zoonotic isolates were enriched for animal-associated β-lactamase genes (e.g., *bla*_TEM-1B_*, bla*_CTX-M-65_), lacked human-associated markers such as *bla*_OXA-1_, and exhibited diverse antimicrobial resistance gene profiles resembling those observed among food-animal isolates. These isolates were also enriched for ColV-associated virulence genes typically linked to avian pathogenic *E. coli*. Putative zoonotic strains contributed substantially to third-generation cephalosporin-resistant UTIs in Quito, Ecuador, challenging assumptions derived from high-income settings that such infections are driven predominantly by human-to-human transmission. These findings highlight the importance of integrated One Health surveillance and mitigation, particularly in low- and middle-income countries where gaps in water, sanitation, and hygiene (WASH) may interact with antimicrobial use in food production to amplify antimicrobial resistance transmission.

**IMPORTANCE:**

ESBL-producing *E. coli* have rapidly emerged as a major global antimicrobial resistance threat. In Latin America, cephalosporins are commonly used in food-animal production, fueling the emergence of ESBL-producing *E. coli*. In low- and middle-income countries, excessive antimicrobial use driven by poorly regulated over-the-counter sales, combined with inadequate water, sanitation, and hygiene (WASH) infrastructure, can facilitate antimicrobial-resistant pathogen transmission from food animals to humans. Using a novel statistical-genomic approach, we found that over one in four cephalosporin-resistant UTIs in Quito, Ecuador, may be caused by *E. coli* strains originating from food animals. Our findings highlight the public health risks associated with antimicrobial use in food-animal production and the role of environmental and infrastructure-related vulnerabilities. As global demand for animal protein continues rising in middle-income countries, controlling zoonotic antimicrobial resistance transmission becomes increasingly urgent for protecting human health through integrated One Health strategies.

## INTRODUCTION

Extended-spectrum β-lactamase-producing *Escherichia coli* (ESBL-producing *E. coli*) are recognized globally as a serious public health threat, with nearly 60,000 attributable deaths in 2019, over 80% of which occurred in low- and middle-income countries (LMICs) (1, 2). ESBL-producing *E. coli* are resistant to third-generation cephalosporins, which are cornerstone antibiotics for managing serious Gram-negative bacterial infections. Infections caused by ESBL-producing *E. coli*, including urinary tract infections (UTIs), are associated with limited treatment options, longer hospital stays, increased costs, and worse clinical outcomes (3). Latin America has been identified as a hotspot for ESBL-producing *E. coli*, with some of the highest reported carriage rates in the world (4–7). However, knowledge gaps remain regarding the burden of community-acquired ESBL-producing *E. coli* infections and their primary drivers of transmission in this region.

The spread of ESBL-producing *E. coli* represents a significant One Health challenge in Latin America, where Ecuador and other middle-income countries are experiencing rapid expansion of livestock production amid gaps in antimicrobial stewardship and water, sanitation, and hygiene (WASH) infrastructure (8, 9). Ecuador, like much of the region, has seen significant growth in large-scale poultry production—the dominant animal protein source—while cattle and swine production remain integral components of a mixed livestock system that includes small-scale farms and informal meat markets (10, 11). This challenge will likely intensify as global demand for animal protein rises—in 2022, Latin America accounted for roughly a quarter of the world’s chicken meat production (12). These conditions are further compounded by incomplete stewardship frameworks in antimicrobial resistance (AMR) control: many countries, including Ecuador, lack integrated One Health approaches in their AMR stewardship, with no explicit inclusion of environmental surveillance and interventions and no actions against the use of antimicrobials as growth promoters (13). Additionally, inadequate WASH infrastructure and informal slaughtering practices create multiple pathways for community exposure to zoonotic pathogens. These structural factors contribute to a high burden of antimicrobial resistance in communities throughout the region.

Despite extensive research on antimicrobial resistance prevalence in either human or animal populations, few investigations have systematically examined the genetic relatedness of ESBL-producing *E. coli* isolates from animals and humans within the same community context. Our previous research in semi-rural communities near Quito found genetically related ESBL-producing *E. coli* strains and mobile genetic elements (MGEs) in children and environmental fecal samples, indicating domestic animals may serve as reservoirs for ESBL genes in the community (14). A follow-up observational study using repeated measures further showed that proximity to commercial food-animal operations and exposure to household animals were associated with increased ESBL-producing *E. coli* carriage among children (15). These findings offer strong evidence of zoonotic transmission and present an opportunity to take the next step: quantifying its contribution to human infections, which is essential for understanding the public health burden and informing targeted mitigation efforts.

Recently, we developed a Bayesian latent class model that leverages the presence and absence of mobile genetic elements associated with particular hosts to predict the underlying source of *E. coli* isolates (16). Unlike phylogenetic approaches that trace individual transmission events, this probabilistic model infers the most likely source of each isolate based on population-level genetic features. The resulting zoonotic fraction reflects the proportion of human infections likely derived from animals. In this study, we applied this model to estimate the zoonotic fraction of UTIs caused by ESBL-producing *E. coli* in a community setting in Ecuador.

## RESULTS

### Host-associated MGEs prevalence in clinical and animal isolates

We screened for 6 human-associated and 11 food animal-associated MGEs in clinical isolates (*n* = 137) and animal fecal *E. coli* isolates (chicken, *n* = 202; pig, *n* = 31; cow, *n* = 10). Upon reviewing the prevalence of host-associated MGEs by host species (Table S1), all food-animal isolates were then aggregated for Bayesian model analyses. A total of 75.3% of human isolates carried at least one human-associated MGE, whereas 95.8% of animal isolates carried at least one food animal-associated MGE. On average, clinical isolates carried 1.5 human-associated and 1.6 food animal-associated MGEs, compared to food-animal isolates that carried on average 0.1 and 3.0 of each type, respectively.

### Proportion of ESBL-producing *E. coli* UTIs attributed to zoonotic strains

**Fig 1:**
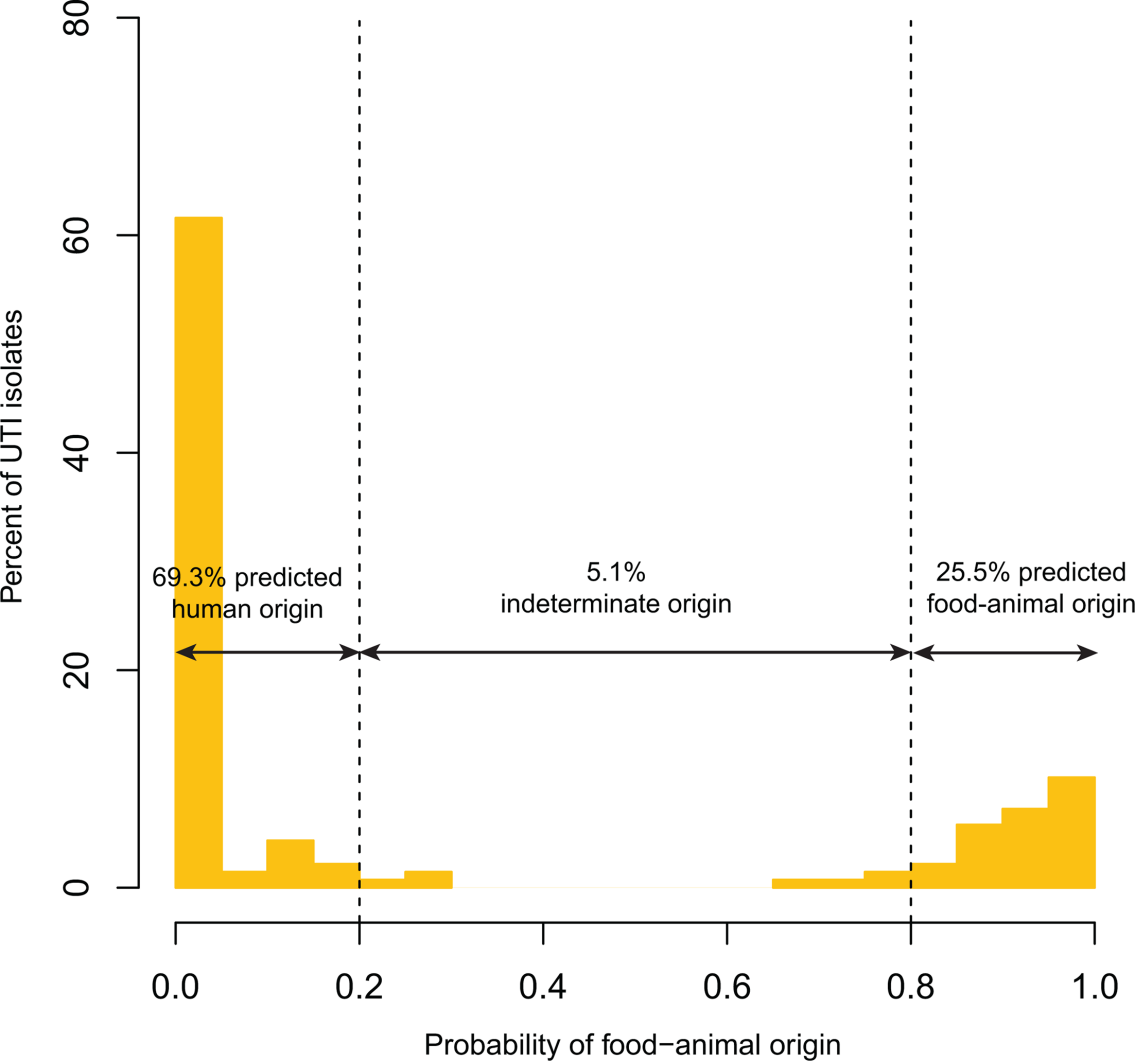
Host origin predictions for human urinary tract infection (UTI) isolates (*n* = 137).

Using the Bayesian latent class model, we estimated that 25.5% (35/137) of clinical ESBL-producing *E. coli* UTI isolates were of likely food-animal origin (≥80% probability; Fig. 1). In contrast, only 1.7% (4/238) of food-animal isolates were inferred to be of human origin (Fig. S1). Isolates were initially categorized into four groups: (i) food-animal isolates (*E. coli* recovered directly from food animals), (ii) zoonotic UTI isolates (≥80% probability of food-animal origin), (iii) human-origin UTI isolates (<80% probability of food-animal origin), and (iv) indeterminate (20%–80% probability of food-animal origin). In subsequent statistical analyses, indeterminate isolates were grouped with human-origin UTI isolates because the indeterminate category represented only a small fraction of samples, limiting statistical power for separate analyses. A large fraction of the ESBL-positive UTI isolates belonged to the pandemic sequence type (ST) 131-*H*30 lineage; however, only 1 of 57 was classified as zoonotic. By comparison, 42.5% (34/80) of non-ST131-*H*30 UTI isolates were zoonotic.

### Sequence types and phylogroups among human and food-animal *E. coli* strains

**Fig 2:**
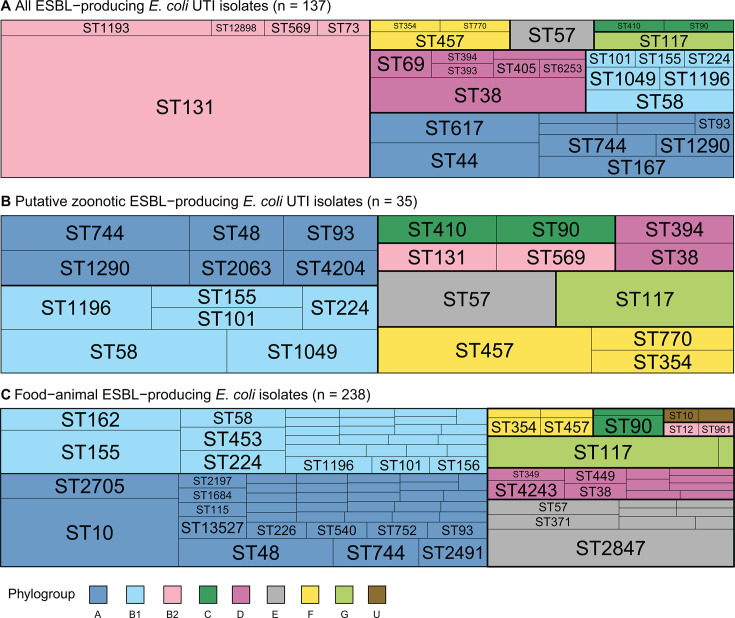
Relative prevalence of sequence types (STs) and phylogroups of *E. coli* isolates. Sequence types are indicated by ST number, and phylogroups are color-coded. Panels show (**A**) all clinical UTI ESBL-producing *E. coli* isolates, (**B**) putative zoonotic UTI isolates, and (**C**) food-animal *E. coli* isolates. STs with fewer than two isolates are not individually labeled.

As mentioned above, multilocus sequence typing (MLST) revealed that nearly half (62/137) of clinical ESBL-producing *E. coli* belonged to ST131, a lineage belonging to the B2 phylogroup and associated with extraintestinal virulence in humans (Fig. 2A). In contrast, most food-animal isolates came from phylogroups A and B1, groups that are often considered commensal or environmental, or phylogroup G, which includes the well-known poultry-associated sequence type ST117(Fig. 2C). The clinical isolates inferred to be zoonotic tended to belong to the same STs and phylogroups as those isolated from food animals (Fig. 2B).

### Genotypic antimicrobial resistance profiles

**Fig 3:**
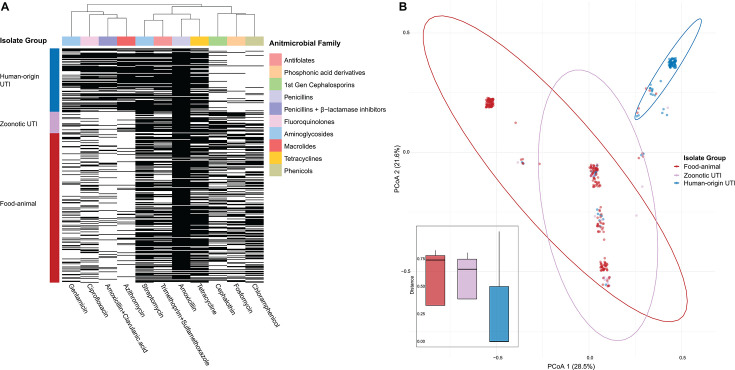
Comparison of antimicrobial resistance genes among *E. coli* isolates. (**A**) Antimicrobial resistance gene profiles. (**B**) Principal Coordinates Analysis (PCoA) of 21 β-lactamase genes, stratified by food-animal isolates (red), zoonotic UTI isolates (purple), and human-origin UTI isolates (blue). Note: Isolates of indeterminate origin were grouped with human-origin UTI isolates. Points in the PCoA plot were jittered to reduce overlap due to high similarity among samples. The inset boxplot shows the distribution of distances to group centroids (multivariate dispersion).

Nearly half of the UTI isolates (*n* = 67, 48.9%) were predicted *in silico* to be resistant to fluoroquinolones (ciprofloxacin), trimethoprim-sulfamethoxazole, and aminoglycosides in addition to third-generation cephalosporins (selected phenotype). Resistance gene profiles differed significantly among isolates from food animals, zoonotic UTI, and human-origin UTI (PERMANOVA, *P* < 0.001). Notably, resistance patterns of zoonotic UTI isolates were more similar to those of food-animal isolates than to human-origin UTI isolates (Fig. 3A). Pairwise comparisons showed that zoonotic strains differed significantly from human-origin isolates in resistance to amoxicillin/ampicillin clavulanic acid, azithromycin, cephalothin, chloramphenicol, ciprofloxacin, and fosfomycin. They also differed significantly from food-animal isolates in resistance to azithromycin, cefepime, gentamicin, and chloramphenicol. Predicted resistance prevalence and pairwise comparisons are detailed in Table S2.

### β-Lactamase gene profile and source-specific associations

We identified a diverse array of β-lactamase genes in both UTI and food-animal *E. coli* isolates. Across all clinical isolates, the most frequently detected β-lactamase genes were *bla*_CTX-M-15_ (78/137), followed by *bla*_OXA-1_ (75/137) and *bla*_CTX-M-3_ (14/137). When stratified by inferred source (Table S3), human-origin UTI isolates were dominated by *bla*_CTX-M-15_ (74/102) and *bla*_OXA-1_ (73/102), while zoonotic UTI isolates most commonly carried *bla*_TEM-1B_ (15/35)—a narrow-spectrum β-lactamase often co-located with ESBL genes—along with *bla*_CTX-M-65_ (12/35) and *bla*_CTX-M-3_ (11/35). Food-animal isolates had substantially greater diversity in β-lactamase gene profiles, with *bla*_CTX-M-55_ (82/238), *bla*_CTX-M-65_ (46/238), and *bla*_TEM-1B_ (38/238) being most prevalent. Indicator analysis confirmed these source-specific associations: *bla*_CTX-M-15_ and *bla*_OXA-1_ with human-origin UTI isolates (*P* < 0.001); *bla*_CMY-2_ with food-animal isolates (*P* = 0.019); and *bla*_TEM-1B_, *bla*_CTX-M-3_, and *bla*_CTX-M-65_ with zoonotic UTI isolates (*P* < 0.001).

### β-Lactamase gene co-occurrence and shared patterns

**Fig 4:**
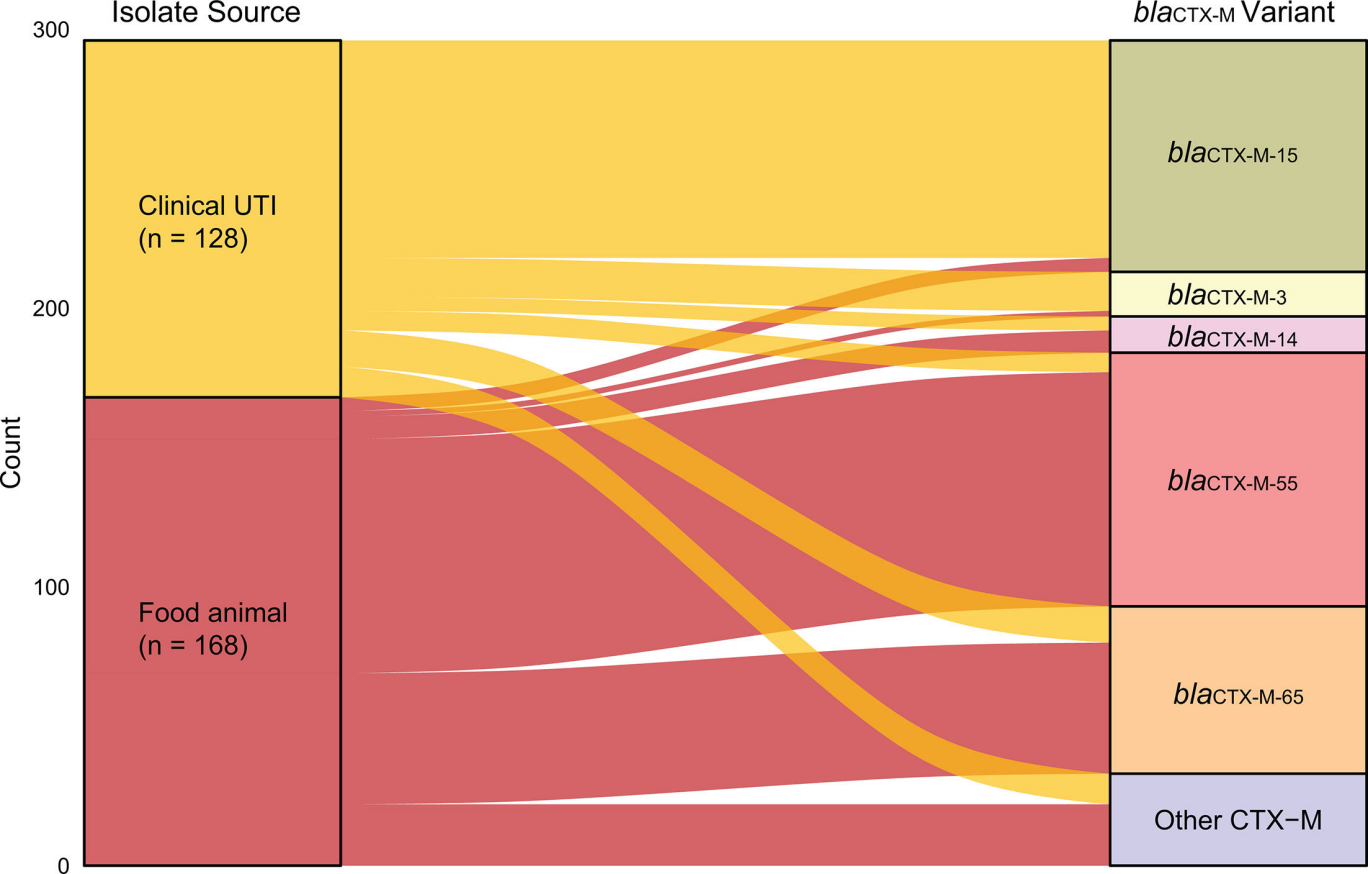
Shared *bla*_CTX-M_ gene variants among ESBL-producing *E. coli* isolates from human UTI and food animals. Not all isolates carried a CTX-M gene.

The combination of *bla*_CTX-M-15_ and *bla*_OXA-1_ (*n* = 64) was exclusively found among human clinical isolates and none were inferred to be zoonotic (Table S3), forming the largest cluster in the β-lactamase gene PCoA plot (Fig. 3B). In contrast, food-animal isolates exhibited greater variety in gene combinations, with the most notable being 20 of 238 isolates carrying *bla*_TEM-1B_ alongside another *bla*_CTX-M_ gene. Particularly, the *bla*_TEM-1B_ plus *bla*_CTX-M-65_ combination was shared between food-animal isolates and zoonotic UTI strains (10/35). Additionally, we observed shared CTX-M gene variants across clinical isolates (both zoonotic and human-origin UTI isolates) and food-animal isolates (Fig. 4). These findings suggest overlapping resistance reservoirs and the potential for animal-to-human transmission of these β-lactamase determinants.

### Virulence factors among the three source groups

The PCoA clustering of the 44 ExPEC-associated virulence genes among human-origin UTI isolates, zoonotic UTI isolates, and food-animal isolates indicated distinct virulence patterns (Fig. S2). Pairwise PerMANOVA comparisons, adjusted with Benjamini-Hochberg corrections, revealed significant differences between human-origin and zoonotic UTI isolates (*P* = 0.003), whereas zoonotic UTI and food-animal isolates were not significantly different (*P* = 0.11). Specifically, human-origin UTI isolates were enriched for classic ExPEC adhesins (*pap*, *afa*, *yfcV*) and broad chromosomal iron uptake systems (*chuA*, *fyuA*, *irp2*, *iucC*), whereas zoonotic UTI and food-animal isolates more frequently carried toxin genes and ColV plasmid-associated genes (*hlyF*, *iroN*, *cvaC*, *etsC*, Table S4). Tests for multivariate dispersion indicated significant differences in group variability (Fig. S2), suggesting that within-group spread may influence observed separations in the PerMANOVA test.

### Phylogenetic relationships

**Fig 5:**
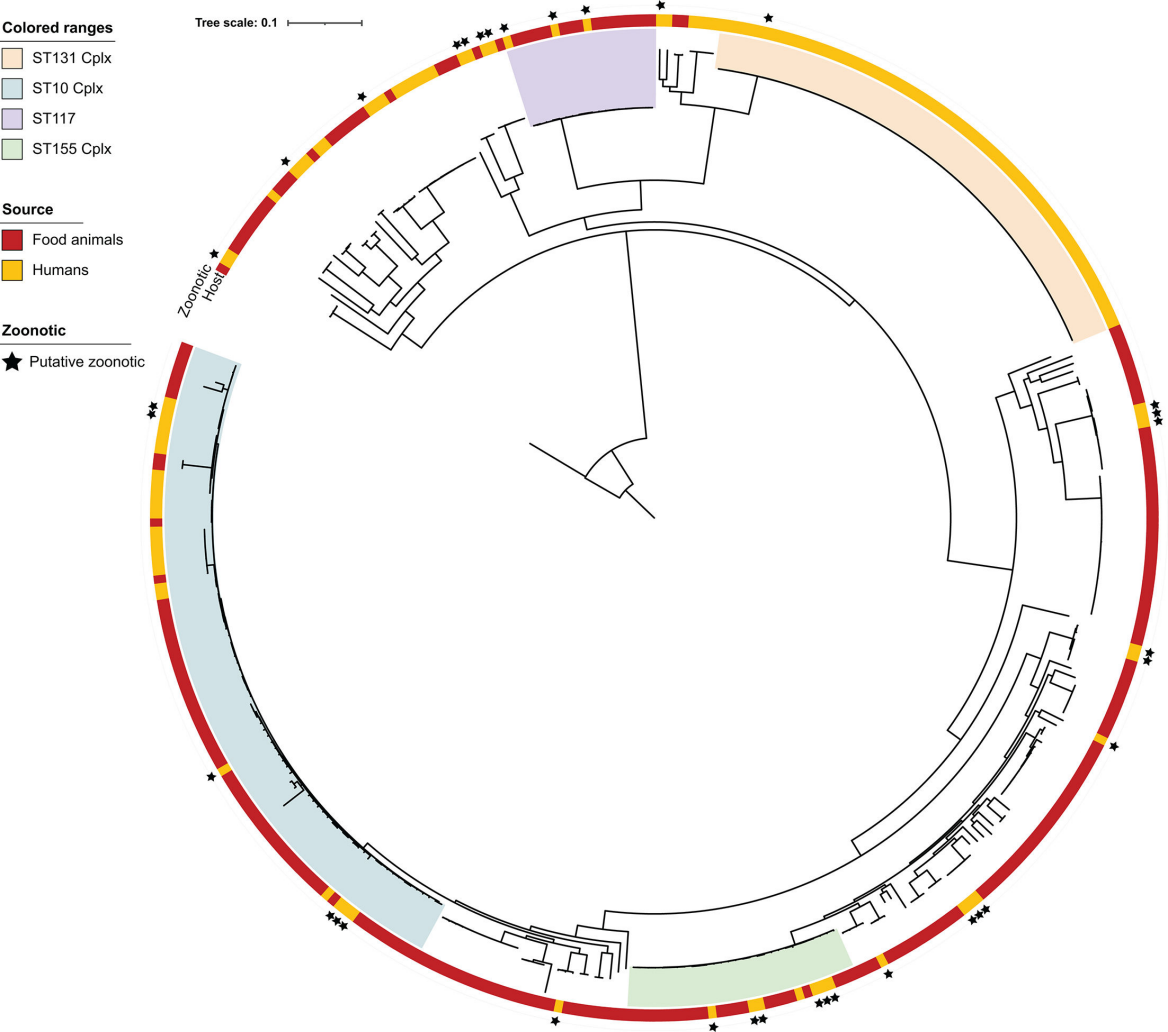
Core-genome phylogeny of ESBL-producing *E. coli* isolates (*n* = 375) annotated with sequence type (ST) and clonal complex (Cplx, color-coded), host source (outer ring), and zoonotic predictions (black stars). This maximum likelihood phylogeny was based on a total of 227,525 core SNPs after purging for recombinations. Scale bar indicates substitutions per site.

The core-SNP-based maximum likelihood phylogenetic tree for all 375 ESBL-producing *E. coli* isolates (Fig. 5) showed that, as expected, ST131 clonal complex clade was composed exclusively of human isolates, consistent with humans being the primary reservoir. In contrast, ST10 complex, ST155 complex (including ST58), and ST117 included both human and animal isolates. Putative zoonotic UTI isolates were distributed across diverse clades and sequence types, suggesting multiple independent transmission events between animals and humans. In many cases, these putative zoonotic UTI isolates clustered closely with food-animal isolates in the core-genome phylogeny, reinforcing their inferred animal origin.

## DISCUSSION

Approximately one-quarter (25.5%) of clinical ESBL-producing *E. coli* UTI isolates in this Ecuadorian population were attributed to food-animal origin. When ST131-*H*30 strains were excluded, the proportion for zoonotic isolates rose to 42.5%, highlighting the significant role food animals may serve as reservoirs of ESBL-producing *E. coli*. This proportion stands in contrast with findings from the United Kingdom, where food- and animal-associated ESBL *E. coli* isolates contributed minimally to human invasive disease (17). They also differ from a Netherlands study of community colonization with ESBL- and pAmpC-producing *E. coli*, which attributed a smaller share of colonization to food and farm-animal sources (18). Studies from the United States have estimated zoonotic contributions between 8% and 18% for *E. coli* isolates not selected for ESBL resistance (16, 19). Although these analyses assess different outcomes (invasive disease vs colonization) across different organism groups, these data suggest that the relative zoonotic contribution to *E. coli* infection in Ecuador appears higher than in high-income settings. The data also suggest that focusing solely on ESBL-producing *E. coli*, which is dominated by human-adapted lineages such as ST131-*H*30, may lead to an underestimation of the relative zoonotic contribution to community-acquired UTIs.

Several contextual factors may help explain the elevated zoonotic burden of ESBL-producing *E. coli* observed in this setting. In Ecuador, direct contact between humans and food animals in semi-rural households with backyard poultry and livestock production creates repeated opportunities for bacterial exchange through shared spaces, environmental contamination, and informal animal husbandry practices. Poultry consumption is another important source of exposure. Poultry remains the primary source of animal protein, with annual per capita consumption of 30–32 kg (10). Poultry production is also an important reservoir for foodborne pathogens, with cephalosporins and other β-lactams commonly used in poultry farming, likely driving the selection of ESBL strains (11, 20). At the same time, insufficient WASH infrastructure amplifies environmental dissemination of resistant bacteria. Across Latin America, only 30%–40% of collected wastewater is treated, and untreated effluent often flows through densely populated areas (21, 22). Together, these factors highlight how rising antimicrobial selection pressure in livestock and frequent local human–animal contact may contribute to sustaining zoonotic transmission of ESBL-producing *E. coli* in the community.

Patterns of ESBL gene carriage were consistent with the broader AMR and phylogenetic findings. Human-origin UTI isolates commonly carried *bla*_CTX-M-15_ and *bla*_OXA-1_, reflecting globally disseminated multidrug-resistant lineages such as ST131-*H*30 (23). In contrast, food-animal isolates frequently carried *bla*_CTX-M-65_ or *bla*_CTX-M-55_, alongside *bla*_TEM-1B_—a combination previously reported in poultry and other food animals (24–27). Notably, 10 (26.6%) zoonotic UTI isolates carried the same *bla*_TEM-1B_ plus *bla*_CTX-M-65_ combination, which has been characterized as being mobilized by the IS26 transposable element (28). This pairing confers resistance to penicillins, first-generation cephalosporins, and β-lactam/β-lactamase inhibitor combinations, suggesting co-selection under widespread antimicrobial use. Although only 1.7% of animal isolates were inferred to be of human origin, the detection of *bla*_CTX-M-15_—a gene predominantly associated with human-adapted lineages—in several animal strains suggests that resistance genes may flow bidirectionally between humans and animals, consistent with similar observations from environmental samples and in other LMICs (29–31).

Food animal production environments in Ecuador appear to foster the evolution and diversification of ESBL-producing *E. coli*. Food animal isolates exhibited high strain diversity, a broad array of ESBL genes, and expanded resistance gene repertoires, consistent with strong antimicrobial selection and widespread horizontal gene transfer. Compared to human isolates, they harbored greater β-lactamase gene diversity, including both CTX-M variants and early-generation β-lactamase genes. Nearly 100 distinct STs were identified among food-animal ESBL-producing *E. coli* in contrast to the dominance of ST131-*H*30 among clinical isolates. This even distribution of STs among food-animal isolates further underscores the genetic heterogeneity present in animal reservoirs (Fig. 2) and highlights the risk for diverse food animals to serve as sources of zoonotic infections.

This genetic heterogeneity extended to virulence profiles. Zoonotic ESBL-producing *E. coli* strains spanned a variety of animal-associated STs and carried distinct virulence gene combinations (Fig. 2; Table S4). For example, three clinical ST58 isolates were inferred to be zoonotic, each carrying the *bla*_TEM-1B_ plus *bla*_CTX-M-65_ combination. All clinical ST117 (*n* = 3) and ST57 (*n* = 3) isolates were also classified as zoonotic; both lineages are well-documented in poultry and are considered to have zoonotic potential (27, 32–34). Several virulence genes, including *hlyF*, *iroN*, *cvaC*, and *etsC*, were prevalent in zoonotic UTI and food-animal isolates. These genes are typically found on ColV-like plasmids associated with avian pathogenic *E. coli* (APEC) (35). Their absence from human-origin UTI isolates and enrichment in zoonotic strains supports the hypothesis that the combination of specific virulence factors may facilitate both colonization in animal hosts and transmission to humans.

This study has several key strengths. First, we applied a novel molecular-epidemiologic model that quantifies the zoonotic burden at the population level, rather than tracing individual transmission events as phylogenetic approaches typically do. This allowed us to estimate the contribution of food animals to ESBL-producing *E. coli* UTIs in Ecuador, a middle-income country with both backyard and commercial livestock production, frequent human-animal contact, and limited wastewater infrastructure. Second, the analysis focused on clinically confirmed infections, enabling direct estimation of the contribution of food animals to human disease, addressing a critical gap in a field that has largely emphasized environmental reservoirs. Third, incorporating regionally representative food-animal genomes allowed the model to better distinguish between human- and animal-associated isolates, enhancing the reliability of zoonotic attribution in this region. Finally, the finding that nearly half of non-ST131 UTI isolates were inferred to originate from food animals challenges the prevailing assumption that ESBL-producing *E. coli* infections are driven predominantly by human-to-human transmission. Together, these strengths make the study a meaningful contribution to our understanding of AMR transmission dynamics in resource-limited settings.

Limitations of this study include the relatively small sample size, the short isolate collection period, and the non-contemporaneous collection of food-animal isolates. Longitudinal genomic surveys suggest that common *E. coli* sequence types can persist for decades in clinical populations although their relative abundances may fluctuate over time (36–38). Importantly, the goal of this study was to quantify zoonotic transmission at the population level rather than to link individual infections to specific animal sources. Because the MGEs used in this analysis are associated with human or animal hosts across multiple sequence types, this temporal separation is unlikely to introduce systematic bias into the zoonotic attribution inferred by the Bayesian latent class model. However, such separation may modestly increase uncertainty.

In addition, the host-associated MGEs used for source attribution were originally identified in U.S. isolate collections and were not selected based on ESBL production. Regional differences in *E. coli* population structure and accessory genome content in Ecuador may, therefore, result in lower observed prevalence of some markers, particularly among animal-associated strains. This could lead to under-recognition of locally circulating zoonotic strains carrying region-specific MGEs not represented in the current marker set. Accordingly, our estimate of zoonotic infections in Ecuador may be conservative.

Lastly, restricting analyses to ESBL-producing isolates may underestimate the contribution of zoonotic *E. coli* to extraintestinal human disease. Selection for ESBL production likely biased the isolate set toward globally disseminated, predominantly anthroponotic lineages, such as ST131-*H*30. Collectively, these factors are likely to bias zoonotic attribution toward conservative estimates, suggesting that the true contribution of food-animal *E. coli* strains to UTIs in Ecuador may be greater than estimated here. Future work could benefit from region-specific marker discovery and contemporaneous, resistance-independent sampling of both human and animal isolates.

In conclusion, our study suggests that food animals are an important reservoir of ESBL-producing *E. coli* responsible for human UTIs in Quito, Ecuador. The convergence of widespread antimicrobial use in both people and food animals (e.g., poultry), combined with inadequate WASH infrastructure, likely drives the emergence and dissemination of diverse ESBL-producing *E. coli* strains. As meat production and antimicrobial use continue to expand, the risk of zoonotic transmission may intensify, highlighting the urgent need for integrated interventions across human and animal health sectors.

## MATERIALS AND METHODS

### Clinical *E. coli* isolates

This study leveraged 137 ceftriaxone-resistant *E. coli* isolates from urinary tract infections that have been described previously (39). Briefly, isolates were obtained from nine outpatient clinics in Quito, Ecuador, between May 2014 and May 2015. Isolates were cultured on Chromocult agar and identified based on β-D-glucuronidase activity. Single colonies were purified in trypticase soy broth, and genomic DNA was extracted using the DNeasy Blood & Tissue Kit (QIAGEN). DNA was sequenced on an Illumina NextSeq 2000 platform.

### Food-animal *E. coli* isolates

A total of 238 ceftriaxone-resistant *E. coli* isolates from animal fecal samples previously collected as part of a repeated measures observational study conducted in seven semi-rural parishes east of Quito, Ecuador, between August 2018 and August 2021 were used in this study (15). Specifically, sampled animals were domestic species (chickens, pigs, and cows) living in close contact with households, such as backyard or small-scale animal keeping. When multiple species were present in a household, one stool sample per species was collected. Fecal samples were cultured on MacConkey agar with 2 mg/L of ceftriaxone, and resistant colonies were confirmed as *E. coli* on Chromocult agar. Genomic DNA was extracted using Wizard Genomic DNA Purification Kit (Promega) and DNeasy Blood & Tissue Kit (QIAGEN) and sequenced on Illumina MiSeq or NovaSeq platforms.

### Host-associated mobile genetic element screening

The details of the 17 previously identified host-associated MGEs can be found in Table S5 (16). The presence or absence of MGEs was determined using MMSeqs2 v17 with ≥80% identity and ≥80% length coverage (40). For 12 elements, an MGE was considered present if at least 1 of its marker genes was detected; for the remaining 5 elements, detection of 2 or more marker genes was required. These calling thresholds were determined by examining gene distribution patterns across isolates to ensure reliable detection while balancing sensitivity and specificity for each element. To support model performance, we curated a context data set and a training set that included human extraintestinal infection isolates from diverse regions and *E. coli* isolates from a variety of food animals.

### MLST, phylogroup, AMR gene, and virulence factor typing

Sequence types of the isolates were determined *in silico* using MLST through EnteroBase, and the *E. coli* phylogroup was determined using Clermont PCR typing (41, 42). Antimicrobial resistance (AMR) genes were screened with ResFinder v4.4.2 at ≥80% coverage and ≥80% identity for genotypic-predicted AMR phenotype analysis and 100% coverage and identity only for *bla_CTX-M_* gene variants (43). Although extraintestinal pathogenic *E. coli* (ExPEC) is the major cause of UTIs, some diarrheagenic strains, such as diffusely adherent and enteroaggregative *E. coli* (DAEC/EAEC), were also reported among UTI cases (44, 45). To capture the pathogenic diversity of UTI-associated *E. coli*, we screened the presence of an extended panel of extraintestinal pathogenic virulence genes using VirulenceFinder v2.0.0 (46) at ≥80% coverage and ≥80% identity.

### Host inference

The host origin of each isolate was inferred using a Bayesian latent class model (47) that integrated both genotype and MGE profiles. Clinical ESBL-producing *E. coli* and animal isolates were assigned to one of three clades; two clades generally associated with human-derived STs and one clade generally housing animal-associated STs. The Bayesian latent class model incorporated the clade along with the pattern of MGE presence/absence to generate probabilistic predictions of host origin for individual isolates. An independent logistic normal prior (mean = 0, SD = 1.5) was applied for latent parameters, and a Beta (1, 1) prior was used for class probabilities (human vs animal). For classification, clinical isolates with a ≥80% probability of food-animal origin were designated as zoonotic, those with ≤20% probability as human origin, and isolates with probabilities between 20% and 80% were considered indeterminate. Indeterminate classifications reflect uncertainty potentially arising from isolates with sparse host-associated MGEs or mixed or conflict signals from human- and animal-associated MGEs.

### Phylogenetic analysis

The phylogenetic tree was constructed using core-genome SNPs, with short reads mapped to the *E. coli* JJ1887 reference genome (GenBank accession no. CP014316), a well-characterized extraintestinal pathogenic *E. coli* urinary isolate, to improve phylogenetic resolution among clinically relevant strains. Read mapping and SNP calling were performed using the NASP pipeline v1.2.0 with BWA-MEM v0.7.12 and GATK v4.2.4.0 (48). Recombinant regions were filtered at 40% using Gubbins v3.3.1 (49). A maximum-likelihood tree was generated using FastTree2 (50) with the GTR model and 1,000 bootstraps and visualized in iTOL v6 (51).

### Statistical analysis

All analyses were conducted in R v4.5.0, and Bayesian inference was performed using JAGS v4.2.0 (52). AMR gene and ExPEC-associated virulence gene prevalence among human-origin UTI isolates, zoonotic UTI isolates, and food-animal isolates were compared using Chi-square test or Fisher’s exact tests with adjustment for multiple comparisons. Antimicrobial resistance gene composition was assessed among groups by PerMANOVA based on Bray-Curtis dissimilarity (R package *vegan*) (53). The ESBL gene indicator analysis was performed using R Package *indicspecies*. ESBL gene and the virulence gene presence patterns were compared using Principal Coordinate Analysis (PCoA) and PerMANOVA global tests based on Jaccard Distance (R package *vegan*). Multiple comparisons were performed with Benjamini-Hochberg corrections.

## Data Availability

Raw sequencing data for all genomes were deposited in the NCBI Short Read Archive (SRA) under BioProject accessions PRJNA1168579 (UTI isolates) and PRJNA861272 (animal isolates). The reference genomes of the 17 host-associated MGEs were deposited under BioProject PRJNA1321378. The details of the 17 previously identified host-associated MGEs can be found in Table S5. The sequence coordinates for each element can be found at the ARAC GitHub repository (https://github.com/araclab/general/tree/main/Food-epidemiology/host_element/minimap2). The data and code that support the findings of this study will be available from the corresponding author upon reasonable request.

## SUPPLEMENTAL MATERIAL

Supplemental material spectrum.03325-25-s0001.docx Tables S1 to S5; Fig. S1 and S2.

ASM does not own the copyrights to Supplemental Material that may be linked to, or accessed through, an article. The authors have granted ASM a non-exclusive, world-wide license to publish the Supplemental Material files. Please contact the corresponding author directly for reuse.
