# Supplementary material for: Investigating the zoonotic origins of ESBL-producing E. coli in community-acquired urinary tract infections in Ecuador
Source: Microbiol Spectr. 2026 Apr 8;14(5):e03325-25. doi: 10.1128/spectrum.03325-25 (PMC13141958; doi:10.1128/spectrum.03325-25)
Supplement: Supplemental material — Tables S1 to S5; Fig. S1 and S2. [file spectrum.03325-25-s0001.docx]

**Supplementary Tables and Figures**

**Investigating the Zoonotic Origins of ESBL-Producing *E. coli* in Community-Acquired Urinary Tract Infections in Ecuador**

**Contents**

Table S1. Prevalence of the 17 host-associated mobile genetic elements (MGEs) across *E. coli* isolates.

Figure S1. Host origin predictions for food-animal *E. coli* isolates (n = 238).

Table S2. Pairwise comparison of *in silico*–predicted antimicrobial resistance (AMR) prevalence among human-origin & indeterminate UTI isolates, zoonotic UTI isolates, and food-animal *E. coli* isolates.

Table S3. Prevalence of β-lactamase genes in human-origin & indeterminate UTI isolates, zoonotic UTI isolates, food-animal isolates.

Table S4. Virulence genes with significantly different prevalence among human-origin & indeterminate UTI isolates, zoonotic UTI isolates, and food-animal *E. coli* isolates.

Figure S2. Principal Coordinate Analysis (PCoA) of 44 extraintestinal pathogenic *E. coli*-associated virulence genes in human-origin UTI, zoonotic UTI, and food-animal *E. coli* isolates.

Table S5. Characteristics of the 17 source-associated mobile genetic elements (MGEs), including MGE type, number of source-associated accessory genes per element, source association, known features and putative selective functions.

**Table S1. Prevalence of the 17 host-associated mobile genetic elements (MGEs) across *E. coli* isolates. MGEs H1–H6 are human-associated, and M1–M11 are food-animal-associated.**

| MGE group | MGEs | Human (n = 138) | Chicken (n = 202) | Pig (n = 31) | Cow (n = 10) |
| --- | --- | --- | --- | --- | --- |
| Human-associated MGEs | H1 | 0.0 | 2.0 | 0.0 | 0.0 |
|  | H2 | 52.2 | 5.9 | 9.7 | 0.0 |
|  | H3 | 0.7 | 1.0 | 0.0 | 0.0 |
|  | H4 | 17.4 | 0.0 | 0.0 | 0.0 |
|  | H5 | 9.4 | 1.5 | 3.2 | 0.0 |
|  | H6 | 67.4 | 0.5 | 0.0 | 0.0 |
| Animal-associated MGEs | M1 | 29.7 | 50.0 | 54.8 | 30.0 |
|  | M2 | 45.7 | 43.1 | 38.7 | 50.0 |
|  | M3 | 22.5 | 30.2 | 48.4 | 50.0 |
|  | M4 | 18.1 | 57.4 | 71.0 | 40.0 |
|  | M5 | 26.8 | 55.9 | 64.5 | 20.0 |
|  | M6 | 3.6 | 35.1 | 35.5 | 10.0 |
|  | M7 | 0.0 | 0.5 | 6.5 | 0.0 |
|  | M8 | 1.4 | 4.5 | 12.9 | 20.0 |
|  | M9 | 2.9 | 6.4 | 16.1 | 0.0 |
|  | M10 | 6.5 | 9.9 | 22.6 | 0.0 |
|  | M11 | 0.7 | 4.5 | 9.7 | 10.0 |

**
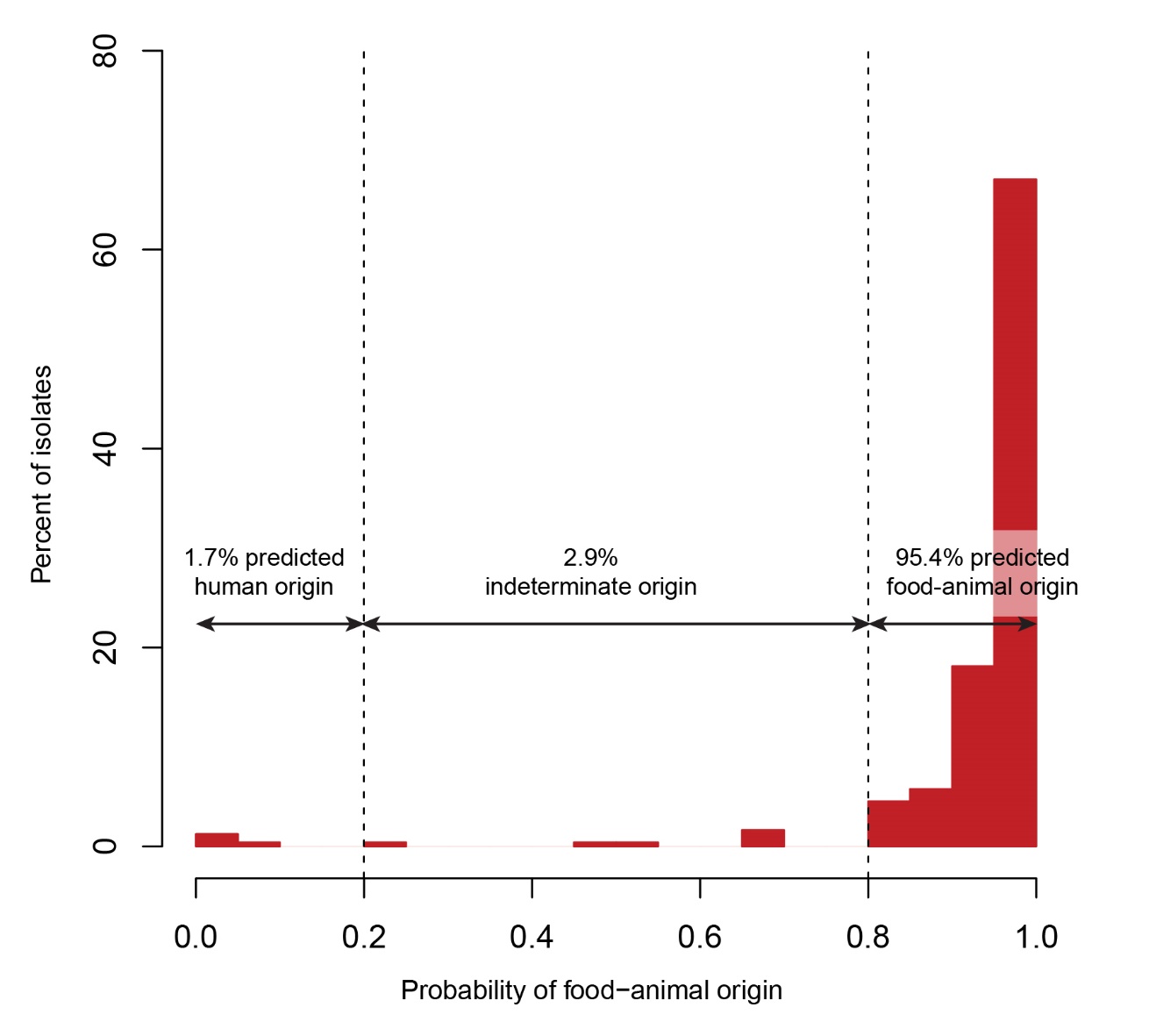
**

**Figure S1. Host origin predictions for food-animal *E. coli* isolates (n = 238).**

**Table S2. Pairwise comparison of *in silico*–predicted** **antimicrobial resistance (AMR) prevalence among human-origin & indeterminate UTI isolates, zoonotic UTI isolates, and food-animal *E. coli* isolates.**

| Antimicrobials | A. Human-origin & indeterminate UTI (n = 102) | B. zoonotic UTI  (n = 35) | C. Food-animal  (n = 238) | Chi-square/Fisher’s exact test pairwise p-value | | |
| --- | --- | --- | --- | --- | --- | --- |
|  |  |  |  | A vs B | A vs C | B vs C |
| Amoxicillin/Ampicillin | 97.1 | 97.1 | 94.2 | 0.505 | 0.128 | 0.702 |
| Amoxicillin/Ampicillin Clavulanic acid | 71.8 | 5.7 | 14.0 | <0.001 | <0.001 | 0.144 |
| Azithromycin | 79.6 | 20.0 | 11.1 | <0.001 | <0.001 | 0.001 |
| Cefepime | 95.1 | 97.1 | 81.1 | 1.000 | <0.001 | 0.015 |
| Cefotaxime/Ceftazidime | 93.2 | 97.1 | 91.4 | 1.000 | 0.343 | 0.749 |
| Cephalothin | 17.5 | 77.1 | 48.6 | <0.001 | <0.001 | 0.124 |
| Chloramphenicol | 9.7 | 40.0 | 58.8 | 0.014 | <0.001 | 0.003 |
| Ciprofloxacin | 73.8 | 22.9 | 34.2 | 0.015 | <0.001 | 0.309 |
| Fosfomycin | 6.8 | 57.1 | 46.9 | <0.001 | <0.001 | 0.922 |
| Gentamicin | 57.3 | 28.6 | 25.5 | 0.365 | <0.001 | 0.012 |
| Streptomycin | 82.5 | 80.0 | 73.3 | 1.000 | 0.173 | 0.275 |
| Sulfamethoxazole | 83.5 | 74.3 | 71.2 | 1.000 | 0.088 | 0.331 |
| Tetracycline | 77.7 | 71.4 | 76.1 | 1.000 | 1.000 | 1.000 |
| Trimethoprim | 79.6 | 71.4 | 58.4 | 0.464 | <0.001 | 0.163 |

**Table S3. Prevalence of β-lactamase genes in human-origin & indeterminate UTI isolates, zoonotic UTI isolates, food-animal isolates. Only genes with prevalence >2% in any group are included.**

| β-lactamase Gene | Human-origin & indeterminate UTI  (n = 102) | Zoonotic UTI (n = 35) | Food-animal  (n = 238) |
| --- | --- | --- | --- |
| *bla*_CTX-M-15_ | 72.5 | 11.4 | 2.1 |
| *bla*_OXA-1_ | 71.6 | 5.7 | 1.3 |
| *bla*_CTX-M-15_ + *bla*_OXA-1_ | 65.7 | 5.7 | 0.0 |
| *bla*_TEM-1B_ | 14.7 | 42.9 | 16.0 |
| *bla*_CTX-M-27_ | 5.9 | 2.9 | 0.0 |
| *bla*_CTX-M-14_ | 3.9 | 2.9 | 3.4 |
| *bla*_CTX-M-3_ | 3.9 | 28.6 | 0.8 |
| *bla*_CTX-M-55_ | 2.9 | 11.4 | 34.5 |
| *bla*_CMY-2_ | 2.0 | 0.0 | 11.3 |
| *bla*_CTX-M-65_ | 1.0 | 34.3 | 19.3 |
| *bla*_TEM-1B_ + *bla*_CTX-M-65_ | 1.0 | 31.4 | 3.4 |
| *bla*_TEM-1B_ + *bla*_CMY-2_ | 1.0 | 0 | 2.5 |
| *bla*_SHV-5_ | 0.0 | 8.6 | 0.0 |
| *bla*_TEM-1A_ | 0.0 | 8.6 | 0.0 |
| *bla*_CTX-M-1_ | 0.0 | 2.9 | 0.4 |
| *bla*_CTX-M-8_ | 0.0 | 2.9 | 5.0 |
| *bla*_SHV-12_ | 0.0 | 0.0 | 4.2 |

**Table S4. Virulence genes with significantly different prevalence among human-origin & indeterminate UTI isolates, zoonotic UTI isolates, and food-animal *E. coli* isolates.**

| Virulence Gene | Human-origin & indeterminate UTI (n = 102) | Zoonotic UTI (n = 35) | Food-animal  (n = 238) | Description |
| --- | --- | --- | --- | --- |
| *afaC* | 17.5 | 2.9 | 0.4 | Afimbrial adhesin |
| *afaD* | 19.4 | 8.6 | 5.0 | Afimbrial adhesin |
| *cea* | 2.9 | 14.3 | 9.7 | Transmembrane toxins |
| *chuA* | 79.6 | 42.9 | 31.9 | Outer membrane hemin receptor (Iron uptake) |
| *cia* | 1.0 | 20.0 | 5.5 | Transmembrane toxins |
| *cib* | 1.0 | 17.1 | 20.6 | Transmembrane toxins |
| *cvaC* | 1.0 | 40.0 | 34.5 | ColV operon (Colonization) |
| *etsC* | 1.0 | 40.0 | 42.9 | Enterobactin transport system |
| *fyuA* | 83.5 | 51.4 | 24.4 | Siderophore receptor (Iron uptake) |
| *hlyE* | 34.0 | 88.6 | 92.0 | Hemolysin E |
| *hlyF* | 1.0 | 57.1 | 54.2 | Hemolysin F |
| *iroN* | 1.9 | 48.6 | 47.5 | Salmochelin operon (Iron uptake) |
| *irp2* | 83.5 | 48.6 | 23.5 | Iron regulated protein 2 |
| *iucC* | 87.4 | 42.9 | 54.2 | Aerobactin synthetase (Iron uptake) |
| *iutA* | 71.8 | 42.9 | 53.8 | Ferric aerobactin receptor (Iron uptake) |
| *kpsE* | 80.6 | 28.6 | 16.8 | Capsule polysaccharide transport |
| *kpsM* | 77.7 | 28.6 | 14.3 | Polysialic acid transport protein |
| *papA* | 63.1 | 14.3 | 10.9 | P fimbriae (Adhesins) |
| *papC* | 53.4 | 22.9 | 16.4 | Outer membrane usher P fimbriae |
| *sitA* | 86.4 | 74.3 | 60.5 | Iron transport protein |
| *usp* | 66.0 | 8.6 | 2.5 | Uropathogenic-specific protein |
| *yfcV* | 65.0 | 17.1 | 2.9 | Fimbrial protein |


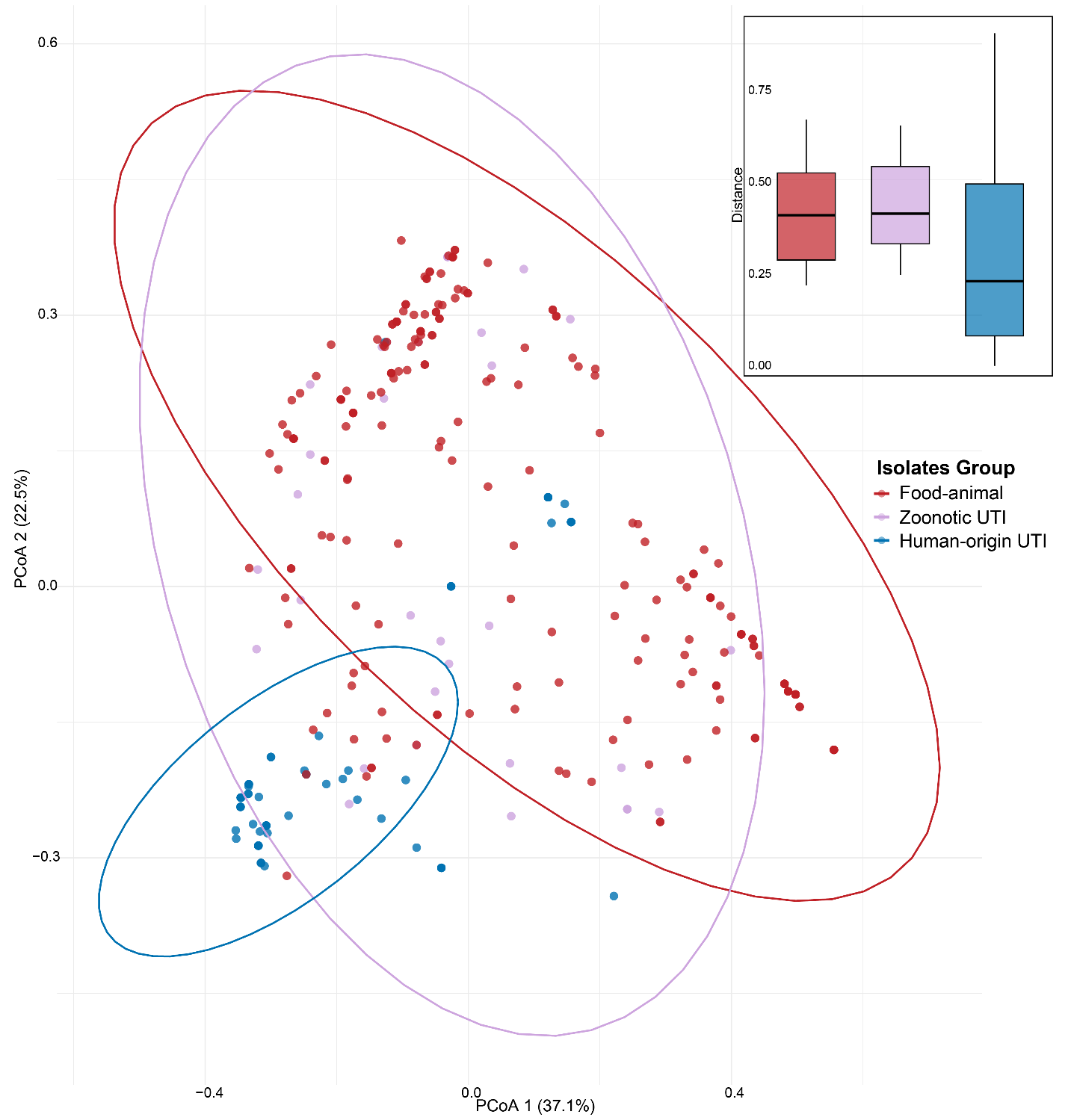


**Figure S2.** Principal Coordinate Analysis (PCoA) of 44 extraintestinal pathogenic *E. coli*-associated virulence genes in human-origin UTI, zoonotic UTI, and food-animal *E. coli* isolates. Note: Isolates of indeterminate origin were grouped with human-origin UTI isolates. Inset boxplot shows the distribution of distances to group centroids (multivariate dispersion).

**Table S5. Characteristics of the 17 source-associated mobile genetic elements (MGEs), including MGE type, number of source-associated accessory genes per element, source association, known features and putative selective functions. Table modified from Liu *et al*., 2023 [1].**

| MGE | No. Genes | Reference Genome (Accession No.) | Associated Host | Putative Selective Function |
| --- | --- | --- | --- | --- |
| H1 | 3 | FMC_2547 (SRR35288285) | Human | Element is three hypothetical proteins, including one with a helix-hairpin-helix DNA binding domain, which might play a role in gene regulation involved in adapting to a human host. In the reference genome, genes are in separate operons with other hypothetical proteins. Adjacent genes include a putative MGE containing the virulence factor tcpC [2]. |
| H2 | 2 | FMC_2547 (SRR35288285) | Human | Needs further study. Element contains two putative phage tail proteins. In the reference genome, these are in an operon with many other phage-related proteins, potentially a prophage. The specific association with human infections is unclear. |
| H3 | 20 | FMC_2624 (SRR35288284) | Human | Element contains all but two genes (clbK and clbQ) from the colibactin biosynthesis cluster, a known virulence factor for UPEC [3,4], along with three additional nearby genes. |
| H4 | 2 | FMC_2547 (SRR35288285) | Human | Needs further study. Contains two conserved hypothetical proteins. Other nearby genes include a putative MobA family protein [5], replication initiation protein, and a third hypothetical protein. |
| H5 | 2 | FMC_2547 (SRR35288285) | Human | Contains a putative MbeB mobilization protein [5] and a hypothetical protein. Nearby are MbeC and rop/rom genes, which regulate plasmid copy number [6]. |
| H6 | 11 | FMC_1856 (SRR35288286) | Human | Cluster includes membrane and periplasmic proteins, including predicted iron and heme transporters [7,8], and a scsC-like copper resistance protein [9,10]. |
| M1 | 11 | FMC_2547 (SRR35288285) | Pork & Chicken | Cluster of phage-related genes. In the reference genome, interrupted by additional phage-related genes, potentially forming a complete prophage. Element splits the lexA repressor [11] from recN [12], possibly affecting SOS response and DNA repair; followed by PasTI toxin-antitoxin pair [13]. |
| M2 | 9 | 57_M1 (SRR35288288) | Chicken | Cluster of phage-related genes. In the reference genome, interrupted by additional phage-related genes, potentially forming a prophage. Element splits xerC and xerD, which may affect gene expression and DNA damage repair [16]. |
| M3 | 41 | FMC_2624 (SRR35288284) | Poultry | Conjugation machinery [17]—enables horizontal gene transfer. In the reference genome, adjacent genes include more conjugation-related genes. |
| M4 | 24 | 57_M1 (SRR35288288) | Poultry | Located on the ColV plasmid [18,19]. Includes colicin E1 and immunity protein [20], VapBC [21], ArtAB [22] toxin-antitoxins [13], mdtH efflux pump [23,24], and iron/heme transport proteins [18]. Genes are dispersed on the plasmid. |
| M5 | 15 | 57_M1 (SRR35288288) | Poultry | Located on the ColV plasmid [18,19]. Includes a putative efflux pump (MacAB-CusC-like) [25,26], a Mig-14-family protein [27], and the colicin V gene [28]. Genes are dispersed on the plasmid. |
| M6 | 3 | 57_M1 (SRR35288288) | Poultry | Located on the ColBM plasmid [29]. Element is a continuous cluster of immunity proteins for colicin M and B and colicin M [28]. Within a larger toxin-antitoxin cluster that includes colicin B [13]. |
| M7 | 4 | 57_M1 (SRR35288288) | Chicken | Located on the ColBM plasmid [29]. Continuous cluster of streptomycin and aminoglycoside resistance genes and groEL/groES chaperone [30]. |
| M8 | 19 | 100_CN_05_B6_M2_C4_P2 (SRR35288287) | Turkey | Copper- and silver-resistance gene cluster [9]. In the reference genome, additional resistance genes are interspersed. |
| M9 | 2 | 100_CN_05_B6_M2_C4_P2 (SRR35288287) | Turkey | Pair of transposases. In the reference genome, separated by another transposase and recombinase, near P fimbriae papC [7] and two neomycin resistance genes—potential impact on gene regulation. |
| M10 | 2 | FMC_2624 (SRR35288284) | Turkey | Contains a LysR-type transcriptional regulator (controls infection-related genes [14]) and sodium-glutamate symporter (stress response [15]). |
| M11 | 6 | 100_CN_05_B6_M2_C4_P2 (SRR35288287) | Turkey | Transposable element, adjacent to M8. |

**Supplementary references**

1. Liu CM, Aziz M, Park DE, Wu Z, Stegger M, Li M, Wang Y, Schmidlin K, Johnson TJ, Koch BJ, Hungate BA, Nordstrom L, Gauld L, Weaver B, Rolland D, Statham S, Hall B, Sariya S, Davis GS, Keim PS, Johnson JR, Price LB. 2023. Using source-associated mobile genetic elements to identify zoonotic extraintestinal *E. coli* infections. One Health 16:100518.
2. Ou Q, Fang J-Q, Zhang Z-S, Chi Z, Fang J, Xu D-Y, et al. TcpC inhibits neutrophil extracellular trap formation by enhancing ubiquitination mediated degradation of peptidylarginine deiminase 4. Nat Commun. 2021;12:3481.
3. Morgan RN, Saleh SE, Farrag HA, Aboulwafa MM. Prevalence and pathologic effects of colibactin and cytotoxic necrotizing factor-1 (Cnf 1) in Escherichia coli: experimental and bioinformatics analyses. Gut Pathog. 2019;11:22.
4. Faïs T, Delmas J, Barnich N, Bonnet R, Dalmasso G. Colibactin: More Than a New Bacterial Toxin. Toxins. 2018;10. <http://dx.doi.org/10.3390/toxins10040151>
5. Francia MV, Varsaki A, Garcillán-Barcia MP, Latorre A, Drainas C, de la Cruz F. A classification scheme for mobilization regions of bacterial plasmids. FEMS Microbiol Rev. 2004;28:79–100.
6. Tomizawa J. Control of ColE1 plasmid replication. Interaction of Rom protein with an unstable complex formed by RNA I and RNA II. J Mol Biol. 1990;212:695–708.
7. Sarowska J, Futoma-Koloch B, Jama-Kmiecik A, et al. Virulence factors, prevalence and potential transmission of extraintestinal pathogenic Escherichia coli isolated from different sources. Gut Pathog. 2019. <http://dx.doi.org/10.1186/s13099-019-0290-0>
8. Subashchandrabose S, Mobley HLT. Virulence and Fitness Determinants of Uropathogenic Escherichia coli. Microbiol Spectr. 2015;3. <http://dx.doi.org/10.1128/microbiolspec.UTI-0015-2012>
9. Cheng G, Ning J, Ahmed S, et al. Selection and dissemination of antimicrobial resistance in Agri-food production. Antimicrob Resist Infect Control. 2019. <http://dx.doi.org/10.1186/s13756-019-0623-2>
10. Shepherd M, Heras B, Achard MES, et al. Structural and functional characterization of ScsC, a periplasmic thioredoxin-like protein from Salmonella enterica serovar Typhimurium. Antioxid Redox Signal. 2013;19:1494–506.
11. Butala M, Zgur-Bertok D, Busby SJW. The bacterial LexA transcriptional repressor. Cell Mol Life Sci. 2009;66:82–93.
12. Keyamura K, Hishida T. Topological DNA-binding of structural maintenance of chromosomes-like RecN promotes DNA double-strand break repair in Escherichia coli. Commun Biol. 2019;2:413.
13. Norton JP, Mulvey MA. Toxin-antitoxin systems are important for niche-specific colonization and stress resistance of uropathogenic Escherichia coli. PLoS Pathog. 2012;8:e1002954.
14. Maddocks SE, Oyston PCF. Structure and function of the LysR-type transcriptional regulator (LTTR) family proteins. Microbiology. 2008;154:3609–23.
15. Feehily C, Karatzas KAG. Role of glutamate metabolism in bacterial responses towards acid and other stresses. J Appl Microbiol. 2013;114:11–24.
16. Rudenko G, Hohenester E, Muller YA. LG/LNS domains: multiple functions – one business end? Trends Biochem Sci. 2001;26:363–8. <http://dx.doi.org/10.1016/s0968-0004(01)01832-1>
17. Cabezón E, Ripoll-Rozada J, Peña A, de la Cruz F, Arechaga I. Towards an integrated model of bacterial conjugation. FEMS Microbiol Rev. 2015;39:81–95.
18. Johnson TJ, Siek KE, Johnson SJ, Nolan LK. DNA sequence of a ColV plasmid and prevalence of selected plasmid-encoded virulence genes among avian Escherichia coli strains. J Bacteriol. 2006;188:745–58.
19. Johnson TJ, Logue CM, Wannemuehler Y, et al. Examination of the source and extended virulence genotypes of Escherichia coli contaminating retail poultry meat. Foodborne Pathog Dis. 2009;6:657–67.
20. Duché D, Issouf M, Lloubès R. Immunity protein protects colicin E2 from OmpT protease. J Biochem. 2009;145:95–101.
21. Winther KS, Gerdes K. Ectopic production of VapCs from Enterobacteria inhibits translation and trans-activates YoeB mRNA interferase. Mol Microbiol. 2009;72:918–30.
22. Littler DR, Ang SY, Moriel DG, et al. Structure–function analyses of a pertussis-like toxin from pathogenic Escherichia coli. J Biol Chem. 2017;292:15143–58.
23. Zhang Y, Dong R, Zhang M, Gao H. Native efflux pumps of Escherichia coli responsible for short and medium chain alcohol. Biochem Eng J. 2018;133:149–56.
24. Yu L, Li W, Li Q, et al. Role of LsrR in the regulation of antibiotic sensitivity in avian pathogenic Escherichia coli. Poult Sci. 2020;99:3675–87.
25. Souabni H, Batista Dos Santos W, Cece Q, et al. Quantitative real-time analysis of the efflux by the MacAB-TolC tripartite efflux pump. Commun Biol. 2021;4:493.
26. Chacón KN, Mealman TD, McEvoy MM, Blackburn NJ. Tracking metal ions through a Cu/Ag efflux pump. Proc Natl Acad Sci U S A. 2014;111:15373–8.
27. McPhee JB, Small CL, Reid-Yu SA, et al. Host defense peptide resistance contributes to colonization and intestinal pathology by E. coli. Infect Immun. 2014;82:3383–93.
28. Cascales E, Buchanan SK, Duché D, et al. Colicin Biology. Microbiol Mol Biol Rev. 2007;71:158–229. <http://dx.doi.org/10.1128/mmbr.00036-06>
29. Johnson TJ, Johnson SJ, Nolan LK. Complete DNA Sequence of a ColBM Plasmid. J Bacteriol. 2006;188:5975–83. <http://dx.doi.org/10.1128/jb.00204-06>
30. Fourie KR, Wilson HL. Understanding GroEL and DnaK Stress Response Proteins as Antigens. Vaccines. 2020;8:773. <http://dx.doi.org/10.3390/vaccines8040773>
